# Cardioneuroablation: Don’t forget atrioventricular node innervation

**DOI:** 10.1016/j.hrcr.2022.10.016

**Published:** 2022-11-04

**Authors:** Jose Carlos Pachon-M, John Clark, Carlos T.C. Pachon

**Affiliations:** ∗ Sao Paulo University, Sao Paulo, Brazil; † Sao Paulo Heart Hospital, Sao Paulo, Brazil; ‡ Sao Paulo Dante Pazzanese Cardiology Institute, Sao Paulo, Brazil; § Akron Children’s Hospital, Akron, Ohio

The authors of the article “Cardioneuroablation: Don’t underestimate the posteromedial left atrial ganglionated plexus”^1^ are to be congratulated for the quality of the clinical case, for a large amount of information and teachings resulting from this report, and, mainly, for disclosing the natural limitations of emerging techniques.

They report a case of a 47-year-old man with vagally mediated syncope due to sinus arrest, who underwent a partial cardioneuroablation (CNA), targeting the superior right and posterior right ganglionated plexi (GP). There was apparent success regarding the sinus node inhibition elimination. However, the atrioventricular (AV) node innervation was not approached. Subsequently, the patient developed an advanced functional AV block (AVB), for which a second procedure was performed, aiming for the left posteromedial GP. The case is also prominent because of the electroencephalogram documentation showing sinus arrest preceding syncope. It is essential to consider that the first CNA was incomplete and, in less than 24 hours, there was a syncope recurrence. However, in a very remarkable opportunity, there was recorded the change from the sinus arrest mechanism to an advanced AVB, very well documented by the authors.

## Cardioneuroablation

CNA is an endocardial radiofrequency catheter ablation on the atrial walls that aims to reduce the long-term vagal tone for treating functional bradyarrhythmias without pacemaker implantation.^2^ In the absence of significant heart disease, the vagus maintains a permanent cardioinhibitory tone so that any vagolytic pharmacological or interventional maneuver promotes an increase in sinus rate and reduction or elimination of functional pauses and AVB.

## CNA technique

Since the initial description, several techniques have been reported.^2^^,^^3^ However, the one most rational and efficient is the stepwise ablation until elimination of the vagal response induced by extracardiac vagal stimulation.^4^ In this way, as the elimination of cardioinhibition is confirmed, one can avoid over-ablation.

## Extracardiac vagal stimulation

Published in 2015, the extracardiac vagal stimulation (ECVS) verifies and measures the vagal effect allowing one to get the best CNA endpoint.^4^ It is performed by placing a catheter in the internal jugular vein near the jugular foramen. At this point, owing to the close proximity, it is possible to stimulate the vagus without direct contact with pulses of 50 Hz / 50 μs / 1 V per kilogram up to 70 V. Typically the response is transitory sinus arrest. Advanced AVB may be observed, mainly when ECVS is performed during atrial pacing. When there is spontaneous or induced AVB, CNA controlled by left vagus ECVS is essential.

## Vagal effect pitfall

Owing to the vast GP interconnection, the two vagus nerves innervate both the sinus node and the AV node. There is, however, a tendency for a greater effect of the right vagus on the sinus node, and a greater effect of the left vagus on the AV node. Both right and left vagus stimulation commonly cause sinus arrest. During asystole, AV conduction cannot be assessed, and therefore AV node inhibition is not revealed. However, the ECVS during atrial pacing typically leads to advanced AVB, showing AV node inhibition also.^4^ Thus, ECVS during atrial pacing shows that the AV node is also inhibited, but the AVB cannot be seen during the absence of atrial activity.

## Strategy for the CNA

During the first CNA for cardioinhibitory syncope, it is crucial to eliminate ECVS-induced asystole as well as the advanced AVB typically revealed by ECVS during atrial pacing.^4^ These two criteria constitute the safest and most rational endpoints. They must be achieved with a good safety margin, as part of the acute CNA effect will disappear owing to natural reinnervation.^5^ Beyond the main four GP areas, it is essential to ablate the P-point in all cases.^2^^,^^6^^,^^7^

## Technical aspects of Extracardiac Vagal Stimulation

In the clinical evaluation of the patient in the article by Ascione and colleagues,^1^ AVB was not observed. Only sinus pauses were recorded, lasting up to 17 seconds at night. ECVS, performed with nonspecific equipment using 30 Hz / 0.5 ms / 25 mA, was probably a limiting factor. These stimulation parameters may be insufficient for 2 reasons:

(1) The pulse width is too long and prevents fast repetitive stimulation of the vagal fibers at the working high frequency during cardioinhibition. It is essential not only to depolarize the fibers, but also to re-stimulate them with high frequency. In the original description, was used 50 μs pulse width, 10 times smaller.^4^
(2) Considering that the impedance in the internal jugular, depending on the catheter, varies from 250 to 800 Ω, the amplitude of 25 mA results in a voltage of 6.25–20 V, far below the ideal stimulation of 1 V / kg up to 70 V. Furthermore, the lack of atrial pacing during effective ECVS is an additional factor contributing to missing the AVB detection. However, as AVB had not been observed, the authors decided to perform a restricted CNA, seeking to treat only sinus node innervation. Nevertheless, syncope recurred in less than 24 hours, but now by an advanced AVB instead of sinus pause. This fact shows that the CNA performed by the authors, while very successful at eliminating sinus cardioinhibition, was not enough to eliminate AV nodal cardioinhibition. Thus, we fully agree with the authors when they state, “don’t underestimate the posteromedial left atrial GP.” In cardioinhibitory syncope, it is recommended that this GP always be ablated in the first procedure.^2^ This type of complication may be prevented by CNA controlled with ECVS because it typically reproduces the AVB during atrial pacing in the electrophysiology lab. With AVB documented, CNA can be expanded until the primary endpoint of vagal effect abolishment over both the sinus and the AV node has been achieved.

## Take-home messages

Take-home messages are as follows:

(1) AV conduction assessment is not possible during sinus pauses. Thus, AV node vagal inhibition cannot be assessed or assumed. However, ECVS can demonstrate that sinus node inhibition is almost always associated with AV node inhibition.
(2) Regardless of the CNA technique, ECVS is essential to control the denervation and to achieve the most rational endpoint for the procedure.
(3) The GP anatomical positions are presumed, and there are significant anatomical variations.
(4) In refractory cases, the atrial fibrillation nest mapping by fractionation or by spectral mapping allows a good tracking in normal heart of the innervation to enlarge the CNA until the vagal effect is abolished, a fundamental endpoint of the procedure.
(5) In CNA for cardioinhibitory syncope, the sinus node and AV node innervations should always be independently treated in the first procedure. They must be considered complementary and absolute endpoints to be achieved with equal commitment.
(6) Owing to the anatomical position and GP interconnections, AV node denervation is more complex and more difficult to be confirmed than denervation of the sinus node.

Finally, we fully agree with the authors that restricted ablation is not enough to treat cardioinhibitory syncope, and complete CNA should always be performed in the index procedure.
